# Sex Cord-Gonadal Stromal Tumor of the Rete Testis

**DOI:** 10.1155/2009/624173

**Published:** 2008-12-31

**Authors:** Kamran P. Sajadi, Rory R. Dalton, James A. Brown

**Affiliations:** ^1^Section of Urology, Department of Surgery, Medical College of Georgia, Augusta, GA 30912, USA; ^2^Department of Pathology, Medical College of Georgia, Augusta, GA 30912, USA

## Abstract

A 34-year-old tetraplegic patient with suppurative epididymitis was found on follow-up examination and ultrasonography to have a testicular mass. The radical orchiectomy specimen contained an undifferentiated spindled sex cord-stromal tumor arising in the rete testis. Testicular sex cord-stromal tumors are far less common than germ cell neoplasms and are usually benign. The close relationship between sex cords and ductules of the rete testis during development provides the opportunity for these uncommon tumors to arise anatomically within the rete tesis. This undifferentiated sex cord-stromal tumor, occurring in a previously unreported location, is an example of an unusual lesion mimicking an intratesticular malignant neoplasm.

## 1. Introduction

Physicians frequently encounter scrotal lesions which, despite a careful history, physical examination, and sonography, remain an enigma. The finding of a discrete mass in the testis requires surgical exploration to exclude a malignant neoplasm. We present a patient whose radical orchiectomy revealed a sex cord-stromal tumor arising within the rete testis, which to the best of our knowledge is a previously unreported location for this uncommon tumor.

## 2. Case Presentation and Management

**Figure 1:**
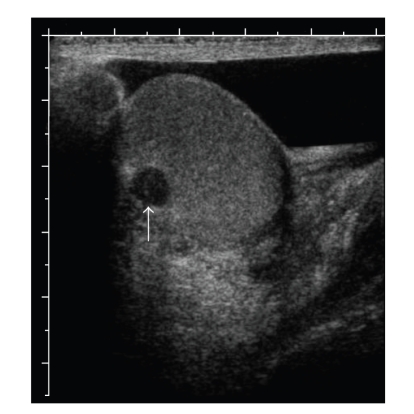
Ultrasound of the right testis, longitudinal view. A hypoechoic lesion (arrow) is seen in the posterior testis, near the mediastinum testis.

A 34-year-old man with C3-C4 tetraplegia presented with right hemiscrotal swelling and purulent drainage from the scrotal skin. Physical examination disclosed a firm right testis mass, a 2 cm scrotal fluctuance, and abscess drainage through the skin at the dependent, posterior aspect of the right hemiscrotum. The abscess had spontaneously opened and drained to a degree that he did not need a former incision and drainage procedure. An ultrasound (Figure 1) revealed an 8 mm × 8 mm × 6 mm intratesticular mass with calcifications. He was treated with ciprofloxacin. Six weeks later, the drainage had ceased and a repeat ultrasound showed the testis mass was stable in size. Preoperative alpha-fetoprotein (AFP), beta- human chorionic gonadotropin (hCG), lactate dehydrogenase, chest X-ray were normal. The decision was made to obtain a CT of the abdomen and pelvis after surgery depending on the pathology results. A right radical orchiectomy was performed. His postoperative course was remarkable only for a small inguinal hematoma which spontaneously drained and resolved.

## 3. Pathology

**Figure 2:**
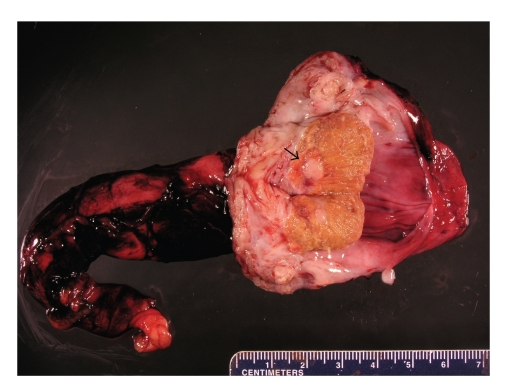
Bisected radical orchiectomy specimen. The tumor can be seen as a smooth, round, pale lesion (arrow) at the mediastinum testis.

The testicle measured 2.5 cm by 2.1 cm and the attached segment of spermatic cord measured 7.3 cm × 2.1 cm × 1.1 cm. The epididymis was firm and slightly diffusely enlarged. A 0.9 cm well-circumscribed, firm tan-white nodule was identified in the testicular hilum (Figure 2). The remainder of the parenchyma of the testicle was unremarkable.

**Figure 3:**
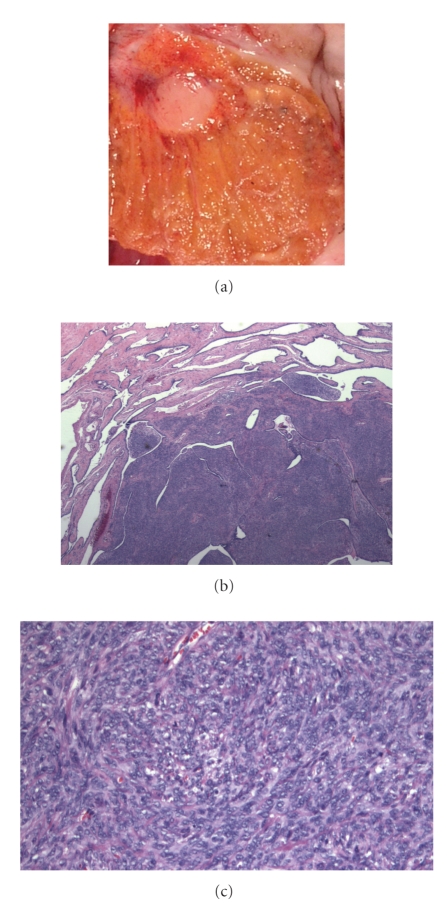
(a) Closer view of gross specimen demonstrating discrete white nodule which was firm. (b) A spindle cell proliferation was intimately associated with unremarkable ductules within the rete testis. (c) Higher power view illustrating bland spindled cell proliferation without mitotic activity.

Microscopic examination (Figure 3, upper right and lower panels) revealed the nodule to be comprised of a well-demarcated, nodular proliferation of bland spindle cells within the mediastinum testis. No areas of tubule or cyst formation were identified. The spindle cells surrounded unremarkable ductules of the rete testis. Neither mitotic activity nor necrosis was present. Immunohistochemical stains for placental alkaline phosphatase (PLAP), CD30, hCG, AFP, CD99, and caldesmon were negative. The spindled cells were positive for vimentin, S-100, WT-1, and focally positive for inhibin and pancytokeratin. Also present were acute and chronic inflammation of the epididymis.

## 4. Discussion

Scrotal lesions are a frequent diagnostic dilemma, and the underlying lesion may remain undefined despite thorough clinical and radiographic investigation. The most important consideration is an underlying testicular germ cell tumor. The close proximity of paratesticular structures, including the rete testis, efferent ductules, epididymis, vas deferens, spermatic cord, tunica, or vestigial testicular appendages, may result in such lesions appearing to be intratesticular on examination and ultrasound [1]. Many such lesions, therefore, require radical orchiectomy to definitively rule out a germ cell neoplasm.

The rete testis develops from sex cords and the degenerating mesonephric duct and includes an intratesticular portion (the tubulae rete and mediastinal rete) and an extratesticular portion comprised of several vesicular structures (extratesticular bullae retis) which form the efferent ductules [2]. As in this case, pathologic processes involving the rete testis may mimic intratesticular germ cell neoplasms clinically and radiographically. Benign processes occurring in the rete testis include epithelial hyperplasia, nodular proliferation of calcifying connective tissue and cysts [3, 4]. Neoplasms occurring in the rete testis include the benign adenoma (also known as benign papillary tumor, cystadenoma, papillary cystadenoma, and adenofibroma) and carcinoma of the rete testis [1, 4]. The carcinoma of the rete—presumably arising from the epithelium of the ductules—is rare and is associated with a poor prognosis [1, 3]. Similar to the confounding picture in this case, patients with adenocarcinoma of the rete testis often present with pain, swelling, and induration resembling epididymo-orchitis [3]. The close relationship during development between the sex cords and the ductules of the rete testis provides the opportunity for SCSTs to arise anatomically within the rete testis [1]. This is supported by the observation that microscopic foci of gonadal stroma are occasionally incidental findings in the extratesticular region of orchiectomy specimens removed for other reasons [1].

Sex cord-stromal tumors (SCSTs) represent approximately 3% of testicular neoplasms and are rare outside the testicle [5]. In the testis, approximately one-half of sex cord-stromal tumors are Leydig cell tumors, with the remainder including Sertoli cell tumors, Granulosa cell tumors, mixed tumors, and tumors of uncertain or indeterminate differentiation (unclassified sex or undifferentiated SCSTs) [6]. Undifferentiated SCSTs are comprised of elements which cannot, at the light microscopic level, be characterized as having Sertoli, Leydig, or Granulosa cell differentiation. Unclassified SCSTs with a predominance of spindle cells have been described in the testis proper [7]. In these cases, immunohistochemistry is useful to establish that the spindle cell proliferation is of sex cord-stromal origin. In a series by Renshaw et al., reactivity for S-100 and smooth muscle actin was consistently present [7]. Interestingly, three of the four unclassified SCSTs they identified arose adjacent to the rete testis. Inhibin is specific for SCSTs and was uniformly reactive in a series of unclassified SCSTs reported by Compérat et al. [6]. In that series, 95% of all SCSTs identified showed immunoreactivity to Vimentin, CD99, and/or inhibin. The immunohistochemical staining pattern in this case was consistent with a sex cord-stromal tumor.

The behavior and prognosis of SCSTs is difficult to predict. As many as 80–90% of these lesions behave in a benign fashion, but they cannot be differentiated from germ cell tumors and their behavior cannot be predicted clinically or sonographically [5]. Features associated with a malignant clinical behavior include large size, invasive margins, vascular invasion, mitotic activity, and necrosis [6, 7]. None of these features were present in our patient, and this undifferentiated spindled SCST is expected to behave in a benign manner.
